# Homeostatically Maintained Resting Naive CD4^+^ T Cells Resist Latent HIV Reactivation

**DOI:** 10.3389/fmicb.2016.01944

**Published:** 2016-12-01

**Authors:** Yasuko Tsunetsugu-Yokota, Mie Kobayahi-Ishihara, Yamato Wada, Kazutaka Terahara, Haruko Takeyama, Ai Kawana-Tachikawa, Kenzo Tokunaga, Makoto Yamagishi, Javier P. Martinez, Andreas Meyerhans

**Affiliations:** ^1^Department of Medical Technology, School of Human Sciences, Tokyo University of TechnologyTokyo, Japan; ^2^Department of Immunology, National Institute of Infectious DiseasesTokyo, Japan; ^3^Department of Life Science and Medical Bioscience, Graduate School of Advanced Science and Engineering, Waseda UniversityTokyo, Japan; ^4^AIDS Research Center, National Institute of Infectious DiseasesTokyo, Japan; ^5^Department of Pathology, National Institute of Infectious DiseasesTokyo, Japan; ^6^Graduate School of Frontier Sciences, University of TokyoTokyo, Japan; ^7^Infection Biology Group, Department of Experimental and Health Sciences, University Pompeu FabraBarcelona, Spain; ^8^Institució Catalana de Recerca i Estudis Avançats (ICREA)Barcelona, Spain

**Keywords:** homeostatic proliferation, HIV, latency, naïve CD4 T cells, cytokines

## Abstract

Homeostatic proliferation (HSP) is a major mechanism by which long-lived naïve and memory CD4^+^ T cells are maintained *in vivo* and suggested to contribute to the persistence of the latent HIV-1 reservoir. However, while many *in vitro* latency models rely on CD4^+^ T cells that were initially differentiated via T-cell receptor (TCR) stimulation into memory/effector cells, latent infection of naïve resting CD4^+^ T cells maintained under HSP conditions has not been fully addressed. Here, we describe an *in vitro* HSP culture system utilizing the cytokines IL-7 and IL-15 that allows studying latency in naïve resting CD4^+^ T cells. CD4^+^ T cells isolated from several healthy donors were infected with HIV pseudotypes expressing GFP and cultured under HSP conditions or TCR conditions as control. Cell proliferation, phenotype, and GFP expression were analyzed by flow cytometry. RNA expression was quantified by qRT-PCR. Under HSP culture conditions, latently HIV-1 infected naïve cells are in part maintained in the non-dividing (= resting) state. Although a few HIV-1 provirus^+^ cells were present in these resting GFP negative cells, the estimated level of GFP transcripts per infected cell seems to indicate a block at the post-transcriptional level. Interestingly, neither TCR nor the prototypic HDAC inhibitor SAHA were able to reactivate HIV-1 provirus from these cells. This lack of reactivation was not due to methylation of the HIV LTR. These results point to a mechanism of HIV control in HSP-cultured resting naïve CD4^+^ T cells that may be distinct from that in TCR-stimulated memory/effector T cells.

## Introduction

The main obstacle to cure an HIV-1 infection is the reservoir of treatment-resistant virus-infected cells. Current antiretroviral therapy (ART) efficiently suppresses HIV replication to undetectable levels in plasma. However, not all of the infected cells are targeted and HIV rapidly rebounds from this reservoir upon treatment interruption (Davey et al., 1999; Chun et al., 2000; Rosenberg et al., 2000; Durand et al., 2012).

The treatment-resistant reservoir consists of latently infected cells that do not produce viral antigens such as infected resting memory or resting naive CD4^+^ T cells (Eriksson et al., 2013; Ho et al., 2013) as well as virus-producing cells that escape drug and immune cell surveillance in sanctuary sites of the lymphatic tissue and the central nervous system (Churchill and Nath, 2013; Fukazawa et al., 2015). Recent evidence also suggests that the HIV integration site into the chromosome can play an important role in provirus expansion and persistence (Maldarelli et al., 2014; Wagner et al., 2014). Estimates on the reservoir size were mainly made from resting CD4^+^ T cells revealing very low total body loads (Chun et al., 1997). Importantly, reservoir assessment by means of virus outgrowth assays and HIV DNA PCR varied by more than two orders of magnitude with less than 1% of proviruses being susceptible to reactivation (Ho et al., 2013). Nonetheless, more than 10% of the HIV proviruses in resting CD4^+^ T cells coded for replication-competent viruses while over 80% were defective. Thus, the present virus outgrowth assays can provide only lower estimates of the total reservoir. The nature of the lack of reactivation of the majority of replication-competent proviruses is unknown and an important issue both for its assessment as part of the persistent reservoir as well as its ability to be targeted by HIV cure strategies.

Studies on the distribution of latent HIV in different CD4^+^ T cell subsets demonstrated an about 10-fold higher infection frequency of memory versus naïve T cells (Ostrowski et al., 1999; Brenchley et al., 2004; Wightman et al., 2010; Josefsson et al., 2013a) and a major contribution of infected central memory and effector memory T cells to the total HIV-1 reservoir (Chomont et al., 2009). Recently, human stem cell-like CD4^+^ memory T cells (Tscm) have been described and identified as a novel HIV-1 reservoir (Buzon et al., 2014; Jaafoura et al., 2014). Tscm cells have several phenotypic markers in common with naïve T cells (Tn) like CD45RA^+^, CD27^+^, CD62L^+^, and IL7R^+^. Although Tn and Tscm cells represent only minor infected T cell subpopulations, they have the longest half-life amongst all infected CD4^+^ T cells (Jaafoura et al., 2014) and can develop into central memory and effector memory T cells upon appropriate stimuli. Thus they represent an important component of the persistent HIV reservoir.

Homeostatic proliferation (HSP) is a major mechanism by which the mature naïve and memory T cell pool is maintained *in vivo* (Surh and Sprent, 2008). The process relies on the interaction of these cells with the cytokines interleukin-7 (IL-7) and interleukin-15 (IL-15) (Boyman et al., 2012), which trigger a signaling cascade that keep T cells, in particular naïve T cells, mostly in a non-dividing state. Such HSP has been suggested to contribute to the persistence of the latent HIV-1 reservoir (Chomont et al., 2009). The study, by Chomont et al. (2009), provided evidence that high level of IL-7 in plasma from HIV-infected aviremic individuals correlated with an increased stability of the HIV reservoir over time. Although it was shown that the plasma IL-15 level was not increased in HIV-infected individuals (Chehimi et al., 1997), it is possible that IL-15 is effective only locally or it is rapidly consumed *in vivo*.

As most *in vitro* latency models rely on CD4^+^ T cells first stimulated via the T-cell receptor (TCR) and differentiated into memory/effector cells, little is known about HIV infection of primary naïve CD4^+^ T cells under homeostatic conditions. To address this, here we used an *in vitro* system of HSP induced by the cytokines IL-7 and IL-15. Under these conditions, primary human CD4^+^ T cells enriched for CD45RA^+^ CD27^+^ can be infected with HIV while maintaining their naïve phenotype. Interestingly, our data suggest that homeostatically maintained latently infected naïve CD4^+^ T cells are refractory to reactivation through T cell receptor signaling or common latency-reversing agents (LRAs). Together this may indicate a distinct mechanism for HIV-1 latency maintenance in cells undergoing HSP.

## Materials and Methods

### Plasmid Preparation

Based on the pNL43-derived GFP-expressing plasmid pNL-E (Yamamoto et al., 2009), we generated a minimal lentivirus (Lenti LTR-GFP) that expresses GFP under the control of HIV-1 LTR. To construct the transfer vector, the pNL-E was digested with NcoI, blunt-ended with T4 polymerase (Roche Diagnostics Inc., GmbH Mannheim, Germany) and further digested with BamHI. The resulting ∼3 kb DNA fragment from 3′ part of env to the end of LTR region of pNL-E containing EGFP-IRES-Nef with a complete 3′ LTR. The fragment was ligated with pCDII-EF-MCS (kindly provided by Dr. Hiroyuki Miyoshi, BioResearch Center, Riken Tsukuba Institute, Tsukuba, Japan) at PmeI and BamHI sites, then the Age I–Age I fragment encoding EF-1α promoter was removed. The resulting Lenti LTR-GFP vector neither encodes Tat nor other accessory proteins of HIV-1. Lenti EF-GFP is the same vector as pCS-CDF-EG, one of the self-inactivating (SIN) vectors developed by Dr. Miyoshi which consists of the *egfp* gene driven by the EF-1α promoter, the rev-responsive element (RRE), the central polypurine tract (*cPPT*), the central termination sequence (CTS) and the Woodchuck hepatitis virus Posttranscriptional Regulatory Element (WPRE). The expression plasmid for the G protein of the vesicular stomatitis virus (VSV), HIV-1 Rev, and the packaging plasmid pCAG-HIV*gag/pol* were also provided by Dr. Hiroyuki Miyoshi. For the pseudotyped HIV-1_NL-E_, Nhe I site of pNL-E was digested, blunt-ended using a Klenow fragment (Roche Diagnostics Inc., GmbH Mannheim, Germany) and re-ligated to generate HIV-1_NL-E_ Δenv.

### Reagents

The histone deacetylase (HDAC) inhibitor SAHA (vorinostat), 2′-deoxy-5-azacytidine (dAzCyt), dimethyl sulfoxide (DMSO), phytohemagglutinin (PHA), interleukin-2 (IL-2), staphylococcal enterotoxin B (SEB) and DNase I were purchased from Sigma-Aldrich (St. Louis, MO, USA). An integrase inhibitor, Raltegravir (RAL) was obtained from Selleck Chemicals (Houston, TX, USA). Purified anti-human CD3 and CD28 were purchased from eBioscience (San Diego, CA, USA).

### Virus Production and Titration

Viruses were prepared as described previously (Yamamoto et al., 2006a). In brief, HEK293T cells in 15 cm dishes were co-transfected with respective plasmids by the calcium phosphate method. Six to eight hours after transfection the culture supernatant was replaced with a fresh DMEM containing L-glutamine, antibiotics, 5 mM MgCls and 100 U of DNase I. Virus-containing supernatant was collected at 40–48 h later, clarified by centrifugation (2500 rpm for 20 min) and kept frozen at -80°C. Virus was concentrated by ultracentrifugation at 28,000 rpm for 2 h at 4°C using SW34Ti rotor and Optima L-90K (Beckman-Coulter, Inc., Fullerton, CA, USA). Gag p24 amounts in supernatants were measured by an in-house HIV-1 Gag p24 enzyme-linked immunosorbent assay (ELISA; Tsunetsugu-Yokota et al., 1995). According to the GFP-expressing frequency in lentivirus infected CEM cells we determined that MOI = 1 was achieved by adding 1 μg of p24 amount to 10^6^ cells.

### Cells and Virus Infection

HEK293T cells and CEM cells were maintained in DMEM and RPMI-1640, respectively, supplemented with 10% heat-inactivated fetal bovine serum (FBS), 100 g/ml penicillin/streptomycin (Invitrogen, San Diego, CA, USA) and 2 mM L-glutamine. Peripheral blood mononuclear cells (PBMCs) were isolated from healthy donors after obtaining written informed consent. Sample collection was approved by the Ethical Committee of the National Institute of Infectious Diseases (NIID, Tokyo, Japan), where the experiment was carried out. Human T cells were first negatively enriched from PBMCs using the EasySep^TM^ Human T cell enrichment kit (StemCell Technologies, Vancouver, BC, Canada). CD4^+^ T cells were further purified from T cells using the EasySep^TM^ human CD4^+^ T cell enrichment kit. In some experiments, naïve CD4^+^ T cells were enriched using the EasySep^TM^ human naïve CD4^+^ T cell enrichment kit (StemCell Technologies, Vancouver, BC, Canada). Purified CD4^+^ T cells were labeled with 1 μM of CFSE or CellTrace^TM^ Violet (Violet tracer) stain kit (Invitrogen, San Diego, CA, USA) in warm PBS at 37°C for 20 min, washed and pulsed with 10% FBS+RPMI for 15 min. For virus infection, cells were either mock infected or infected with lentiviruses by spinoculation at room temperature, 2800 rpm for 2 h (Swiggard et al., 2005). If not stated otherwise, cells were infected with viruses at a MOI of around 1. Cells were washed 3 times and cultured in 10% FBS+RPMI supplemented with 5% human serum, IL-7 and IL-15 (PeproTech, London, UK) at 10 ng/ml each. A part of cells were activated using plate-coated anti-CD3 mAb (5 μg/ml) and anti-CD28 mAb (1 μg/ml) in 10% RPMI containing 40 U IL-2. In some experiments, monocyte-derived dendritic cells (MDDC) from the same donor were prepared as described previously (Yamamoto et al., 2009), pulsed with SEB antigen at 100 ng/ml, and cocultured with CD4^+^ T cells.

### RNA Extraction and Quantitative RT-PCR

RNA was extracted using PureLink RNA Mini Kit (Life Technologies, Carlsbad, CA, USA), and cDNA was synthesized by using SuperScript VILO Master mix (Life Technologies, Carlsbad, CA, USA) according to the protocol by the company. The cDNA product was then mixed with 10 μl of polymerase mixture (Eagle Taq Master Mix kits: Roche Diagnostics Inc., GmbH Mannheim, Germany), 0.5 μl each of 10 μM primers and 5 μM TaqMan probe in a total volume of 20 μl. The PCR was performed using the Mx3000P qPCR system (Stratagene, La Jolla, CA, USA). The PCR reaction was set as 40 cycles of 60°C for 15 s, and 95°C for 15 s after a 10 min denaturing step at 95°C. The following primers and probes for a TaqMan assay were used: EGFP-2F, 5′-gaccactaccagcagaacac-3′, EGFP-2R, 5′-gaactccagcaggaccatg-3′, probe, [6-FAM] agcacccagtccgccctgagca [BHQ-1], RNase P-F, 5′-agatttggacctgcgagcg-3′, RNase P-R, 5′-gagcggctgtctccacaagt-3′, probe, [6-FAM] ttctgacctgaaggctctgcgcg [BHQ-1]. For the detection system by Sybr Green, cDNA was mixed with 10 μl of Platinum Taq two-step Sybr qRT-PCR solution (Invitrogen, San Diego, CA, USA), 0.5 μl each of 10 mM primers and 0.5 μl of Rox dye in a total volume of 20 μl according to the protocol from the company. First incubation for 30 min at 50°C, followed by 40 cycles of 15 s at 95°C, 15 s at 60°C and 15 s at 70°C and finally 5 s at 60°C and 5 s at 95°C. The primers used were: Hs-SAMHD1-F, 5′-tcgtccgaatcattgatacacc-3′, Hs-SAMHD1-R, 5′-gtgcgtgaactagacatcctg-3′, hA3G-rtp-S, 5′-cgcagcctgtgtcagaaaag-3′, hA3G-rtp-A, 5′-ccaacagtgctgaaattcgtcata-3′. EF-1α 294F, 5′-gaacagttgggtcgctttgctgttc-3′, EF-1α 307R, 5′-gacacccaccgcaactgtct-3′. The primer sets of EF-1α and RNase P were used for the endogenous gene expression as a reference.

### HIV Integration Assay

Genomic DNA from collected cell samples was extracted using a QIAamp DNA Micro Kit (Qiagen, Valencia, CA, USA). The copy number of integrated viral DNA was measured according to the real-time nested *Alu*-HIV PCR assay (Yamamoto et al., 2006b) with some modifications. During the first-round PCR, *Alu*-gag sequences were amplified in a 25 μl reaction mixture containing extracted DNA, 1x High Fidelity PCR buffer (Invitrogen, San Diego, CA, USA), 0.2 mM mixed dNTPs, 2 mM MgSO_4_, 200 nM of the Alu-1 primer (5′-tcccagctactggggaggctgagg-3′; Brussel and Sonigo, 2003), 200 nM of the Alu-2 primer (5′-gcctcccaaagtgctgggattacag-3′; Brussel and Sonigo, 2003), 200 nM of the first-gag-R primer (5′-caatatcatacgccgagagtgcgcgcttcagcaag-3′), and one unit of a Platinum *Taq* DNA Polymerase High Fidelity (Invitrogen San Diego, CA, USA). The first-round PCR condition was as follows: a DNA denaturation step of 2 min at 94°C and then 20 cycles of amplification (94°C for 30 s, 50°C for 30 s, 68°C for 100 s) on a TaKaRa PCR Thermal Cycler Dice (Takara, Japan). Note that the first-round PCR efficiency is affected by the number of *Alu* sites (Agosto et al., 2007). Therefore, the amount of total DNA was adjusted to be 1 × 10^5^ copies of β-globin to maintain the number of *Alu* sites. If the amount of sample DNA was <1 × 10^5^ copies of β-globin, DNA extracted from CEM cells was added. The copy number of β-globin was determined by real-time PCR using a primer set (forward, 5′-caagaaagtgctcggtgcctt-3′; reverse, 5′-cctgaagttctcaggatccacg-3′) and a probe (FAM-5′-acactgagtgagctgcactgtga-3′-BHQ-1) with the Mx3000P qPCR System (Stratagene, Amsterdam, The Netherlands). The second-round PCR was performed in a 25 μl reaction mixture containing 1 μl of the first-round PCR product, 1x EagleTaq Master Mix (Roche Diagnostics Inc., GmbH Mannheim, Germany), 100 nM of the U5 primer (5′-ccgtctgttgtgtgactctgg-3′; Suzuki et al., 2003), 100 nM of the second-tag-R primer (5′-caatatcatacgccgagagtgc-3′; Suzuki et al., 2003), and 50 nM of the second PCR probe (FAM-5′-cgcttcagcaagccgagtcctgc-3′-BHQ-1). The second-round PCR condition was as follows: a DNA denaturation and polymerase activation step of 10 min at 95°C and then 40 cycles of amplification (95°C for 15 s, 60°C for 1 min) on a Mx3000P qPCR system. A standard curve was prepared by using a serial dilution of DNA extracted from ACH-2 cells, which contain one copy of HIV-1 provirus per genome.

The amount of integrated viral DNA per cell was calculated by using the copy number of β-globin (two copies per diploid cell) as reference. For the calculation of a provirus copy number, a part of CD4^+^ T cells were pretreated with RAL for 30 min and infected in the presence of RAL to discriminate the genuine integration from contaminating unintegrated viral DNAs.

### Methylation Analysis

CpG DNA methylation within the LTR was analyzed by bisulfite sequencing. Genomic DNA from the each sorted population was subjected into MethylEasy Xceed Rapid DNA Bisulphite Modification kit (Human Genetic Signatures, NSW, Australia). PCR primers for the modified DNA were as follows: Fw, 5′-tttgttatattttgtgagtttgtat-3′; Rv, 5′-acaactacttatatacaacatcta-3′. Resulting amplicons were TA-cloned into the pGEM-T easy vector (Promega), followed by DNA sequence analysis.

### Flow Cytometry

The markers for T-cell activation and proliferation were analyzed by a FACScanto II (BD Biosciences, Heidelberg, Germany) flow cytometer. Antibodies used in this study were: PerCP-labeled anti-human CD4 (clone RPA-T4), PE-Cy7 labeled anti-human CD45RA (clone HI100), Alexa Fluor 700-labeled anti-CD27 (clone O323), FITC-anti-human CD25 (BC96), all purchased from (BioLegend, San Diego, CA, USA). Alexa Fluor 647-labeled anti-human CD11a (LFA-1: TS1/22.1.1.13) was prepared from hybridoma cells (ATCC No. HB 202) and conjugated using Alexa Fluor succinimidyl esters (Invitrogen, Carlsbad, CA, USA). Dead cells were stained with a LIVE/DEAD Fixable Dead Cell Stain Kit (L34957, Invitrogen, Carlsbad, CA, USA) and were gated out during analysis. For cell sorting, a FACSaria II cytometer was used. The data were analyzed by FACSdiva software and reanalyzed by Flowjo ver 8.8.7 (Tree Star Inc., CA, USA).

### Statistical Analysis

For the statistical analysis, Prism software ver. 5.0 (GraphPad Software Inc., San Diego, CA, USA) was used. The non-parametric two-tailed paired *t*-test (Wilcoxon matched-pairs signed rank test) was applied for the evaluation of statistical difference of two groups.

## Results

### Homeostatic Proliferation Maintains the Phenotypic State of Naïve CD4^+^ T Cells

**FIGURE 1:**
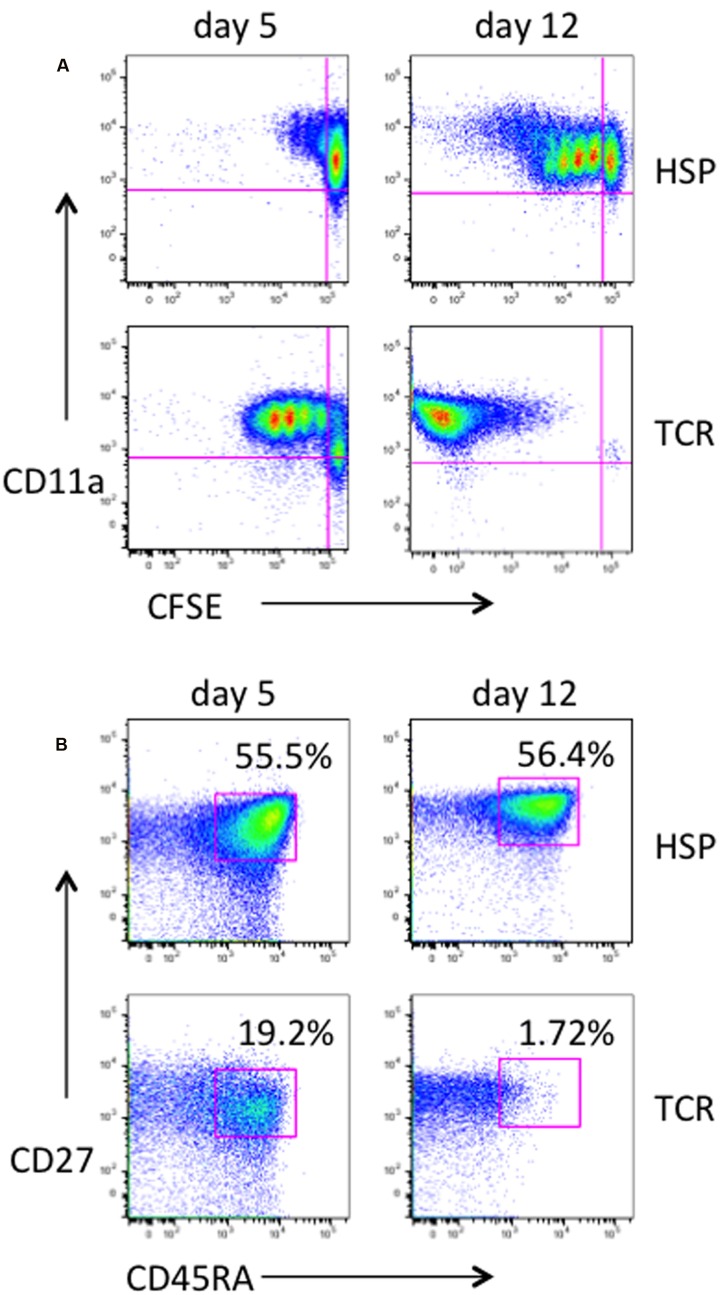
**Characterization of homeostatic proliferation of primary human CD4^+^ T cells.** Primary CD4 T cells were purified from PBMC of healthy donors, labeled with CFSE and cultured under homeostatic conditions with 10 ng/mL of both IL-7 and IL-15 (HSP culture). As controls, CD4 T cells were stimulated with plate-coated anti-CD3 mAb at 5 μg/mL and 1 μg/mL anti-CD28 mAb, and kept in culture in the presence of 40U IL-2 (TCR culture). At days 5 and 12, cells were collected, stained with PerCP-CD4, PE-Cy7-CD45RA, Alexa Fluor 700-CD27, and Alexa Fluor 647-CD11a, and analyzed by flow cytometry. Live and CD4-positive cells were gated. Dot plots of CD11a expression against CFSE are shown for HSP culture and controls **(A)**. The majority of cells under homeostatic culture conditions as opposed to the TCR-stimulated control population remained in a naive phenotype as judged by CD27 and CD45RA expression **(B)**. Percentages of naïve cells are given.

*In vivo* the T cell pool under cytokine-driven homeostasis remains largely unchanged (Chomont et al., 2009). To characterize the proliferation and phenotype status of T cells undergoing HSP, primary purified CD4^+^ T cells were cultured in the presence of both IL-7 and IL-15 (designated here as HSP culture) or stimulated via the TCR using immobilized anti-CD3 and anti-CD28 in the presence of IL-2 (designated as TCR culture) as control. We considered CD45RA^+^/CD27^+^ CD4^+^ T cells as naïve and CD45RA^-^/CD27^+^ as early memory cells. Under HSP culture conditions, CFSE-labeled non-dividing ( = resting) CD4^+^ T cells were activated (CD11a expression) and the cells were able to gradually proliferate (**Figure 1A**, upper panel), whereas under TCR culture conditions most of the cells proliferated vigorously and lost the CFSE dye at day 12 (**Figure 1A**, lower panel). Similarly to a previous report by Unumatz et al. (1999), the proportion of resting CD4^+^ T cells with a naïve phenotype was maintained during the time of HSP culture (**Figure 1B**). This pattern of HSP was observed in several donors (data not shown) and make HSP culture a suitable system to analyze the natural process of HIV infection of naïve CD4^+^ T cells *in vitro*.

### HSP and TCR Culture Conditions Differentially Affect HIV LTR-Dependent Expression of a GFP Marker Gene in Infected CD4^+^ T Cells

**FIGURE 2:**
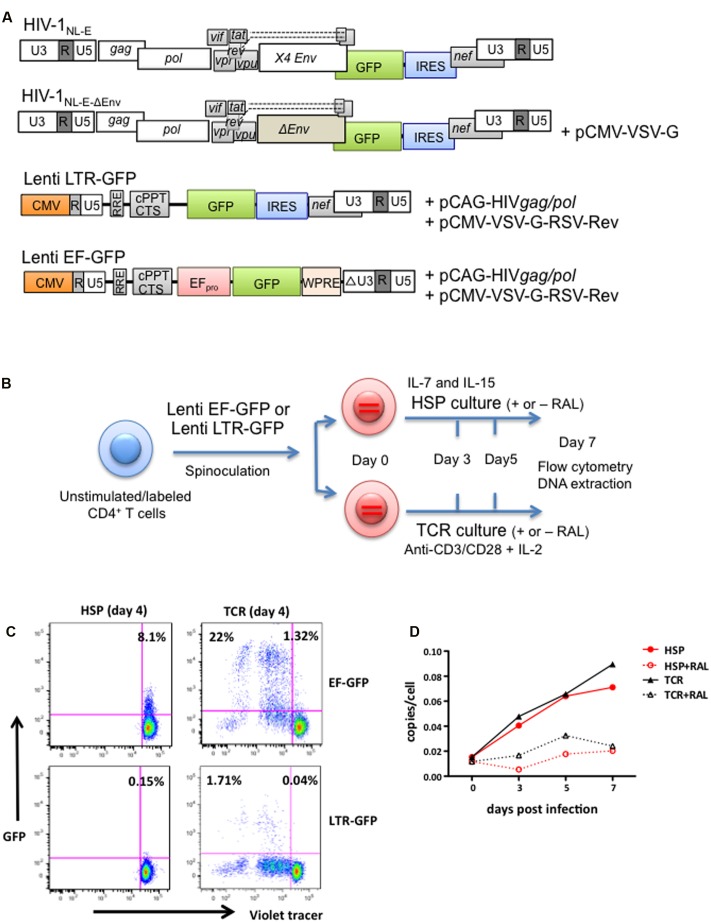
**HIV LTR-dependent expression of a GFP marker gene is differentially influenced by HSP and TCR culture of lentivirus-infected CD4^+^ T cells.** **(A)** Structure of the parental GFP-expressing HIV-1_NL-E_ vector and its derivatives used in this study: HIV-1_NL-E-ΔEnv_, Lenti LTR-GFP (encoding LTR-driven GFP) and Lenti EF-GFP (encoding EF-1α promoter-driven GFP). Plasmid names used for co-transfection for virus production are given. See “Materials and Methods” for details on vector generation. **(B)** Experimental strategy outline. Purified, unstimulated, Violet tracer-labeled CD4^+^ T cells were spinoculated with Lenti LTR-GFP or Lenti EF-GFP, and cultured under HSP or TCR conditions. **(C)** EF promotor- or LTR-driven marker gene expression under HSP culture or TCR conditions. Infected and cultured cells were analyzed for GFP expression and proliferation (Violet Tracer) at day 4 by flow cytometry (HSP culture; left panel and TCR culture; right panel). A representative plot from four different donors is shown. **(D)** Lentivirus-infected CD4^+^ T cells show similar levels of vector integration under HSP and TCR culture conditions. CD4^+^ T cells were infected as under **(B)**, cultured in the presence or absence of integrase inhibitor Raltegravir (RAL) and collected at days 0, 3, 5, and 7. The level of vector integration was analyzed by Alu-PCR. The copies per cell are shown for all time points tested.

The structures of all HIV-1 pseudotype viruses (HIVpp) used in this study are illustrated in **Figure 2A**. First, in order to analyze HIV-1 infection under HSP conditions, we infected CD4^+^ T cells with two minimal lentiviral vectors encoding a GFP marker gene under the control of either the HIV-1 LTR (Lenti LTR-GFP) or the EF-1α promoter (Lenti EF-GFP) (**Figure 2A**). The experimental strategy is outlined in **Figure 2B**. Briefly, unstimulated CD4^+^ T cells purified from blood of healthy donors were labeled with a Violet tracer dye, and infected with Lenti LTR-GFP or Lenti EF-GFP pseudoviruses by spinoculation. Infected cells were cultured under HSP or TCR culture conditions, and both the proliferation and GFP expression were monitored at days 0, 4, and 7 by flow cytometry. **Figure 2C** shows the proliferation and GFP expression profiles at day 4. Around 20% of Lenti EF-GFP-infected CD4^+^ T cells expressed GFP in TCR cultures, whereas only 8.1% of them expressed GFP under HSP conditions (**Figure 2C**, upper panels). However, the percentage of GFP-expressing CD4^+^ T cells in HSP culture increased to 24.6% at day 7 (data not shown), suggesting that HIVpp transcription progresses slowly in these cells. In comparison, the frequency of GFP-expressing cells in Lenti LTR-GFP-infected CD4^+^ T cells was lower in both culture conditions with a difference of around 10-fold between HSP (0.15%) and TCR conditions (1.75%) (**Figure 2C**, lower panel). To assess whether the lower frequency of GFP^+^ cells after Lenti LTR-GFP infection in HSP compared to the TCR culture could be attributed to distinct integration efficiencies in the two culture systems, we analyzed by Alu-PCR proviral copy numbers of Lenti LTR-GFP-infected CD4^+^ T cells at days 0, 3, 5, and 7 after culturing under HSP or TCR conditions in the presence or absence of the HIV integrase inhibitor RAL. As previously described (Swiggard et al., 2005), the integration process progressed slowly in primary CD4^+^ T cells. **Figure 2D** shows the results representative of seven donors. We observed no clear differences in the provirus copy numbers per cell basis between the two culture conditions. Our values are in the same range as those found by others and are in the order of 60-fold higher than seen in patient samples (Chomont et al., 2009; Buzon et al., 2014). The presence of RAL efficiently reduced the copy number in each time point, indicating that genuine integration efficiency was monitored. Taken together, these results indicate that the HIV-1 LTR is mostly restricted at a transcriptional level in both HSP and TCR conditions, with HSP-cultured CD4^+^ T cells receiving stronger restriction than TCR-cultured cells.

### Lack of GFP Expression from Infected Resting CD4^+^ T Cells upon TCR Stimulation Is Not Due to SAMHD1- or APOBEC3G-Mediated Restriction

**FIGURE 3:**
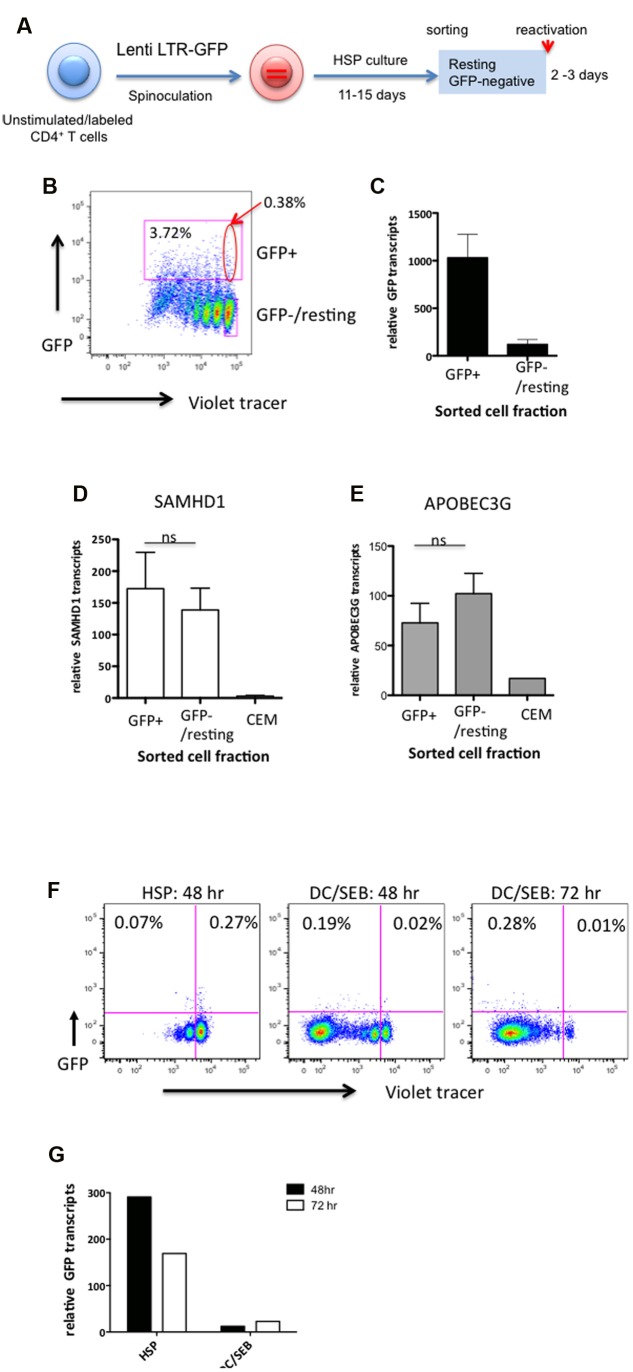
**The impairment of the HIV marker gene expression (GFP) in HSP-cultured resting CD4^+^ T cells is not linked to increased levels of SAMHD1 or APOBEC3G, and is not induced upon TCR engagement by antigen-pulsed dendritic cells.** **(A)** Outline of experimental strategy. Violet tracer-labeled CD4^+^ T cells were infected with Lenti LTR-GFP and cultured under HSP conditions for 11–15 days. GFP-positive and resting GFP-negative cells were sorted by preparative flow cytometry as marked in the analytical dot plot **(B)**. Total RNA from both cell populations was isolated, reverse transcribed with random hexamers and subjected to quantitative PCR specific for GFP **(C)**, SAMHD1 **(D)**, and APOBEC3G **(E)** transcripts. RNA isolated from CEM cell lysates was used as control for SAMHD1 and APOBEC3G transcripts. Transcript levels were normalized to endogenous EF-1α **(C)** or RNase P **(D,E)** mRNA levels. For panels **(F,G)**, resting GFP-negative cells were sorted by preparative flow cytometry and cultured in HSP conditions (HSP), or the presence of SEB-pulsed DCs (DC/SEB). Cell proliferation (Violet tracer) and GFP expression were analyzed by flow cytometry after 48 or 72 h **(F)**. GFP transcripts were analyzed by quantitative RT-PCR from isolated total RNA **(G)**. Transcripts levels were normalized to endogenous EF-1α mRNA levels.

To analyze HIV transcriptional activity in cells undergoing HSP, CD4^+^ T cells were infected with Lenti LTR-GFP and cultured under HSP conditions as depicted in **Figure 3A**. Eleven to fifteen days after infection, total GFP^+^ cells (**Figure 3B**, upper square) and GFP^-^/resting cells (**Figure 3B**, lower square) were sorted by flow cytometry. GFP mRNA transcript levels in the sorted cell populations were analyzed by qRT-PCR. **Figure 3C** shows the levels of GFP transcripts relative to that of endogenous EF-1α transcripts from sorted GFP^+^ and GFP^-^/resting cells (mean of five different donors). Although the GFP mRNA levels in the GFP^+^ fraction was around 10-fold higher, GFP transcripts from GFP^-^/resting CD4^+^ T cells were also detectable. These latter cells harbored an estimated 0.012 to 0.032 proviral copies per cell (mean = 0.02, *SD* = 0.01, *n* = 5). Supposing a proviral copy number of one in the GFP^+^ fraction (**Figure 3C** left column), roughly 50-fold less provirus-positive cells should be present in the GFP^-^/resting fraction (**Figure 3C** right column). Based on the determined transcript levels, this would correspond to a fivefold higher GFP transcript level per provirus containing GFP^-^/resting cell. Nonetheless, there was no GFP expression detectable by flow cytometer. This may be indicative of a translational block of provirus expression in GFP^-^/resting CD4^+^ T cells.

To investigate whether the apparent low level of HIV transcriptional activity observed in resting HSP cultured cells was correlated to an upregulation of the restriction factors SAMHD1 and/or APOBEC3G (Chiu et al., 2005; Laguette et al., 2011) we quantitated transcripts of these genes in the sorted GFP^+^ and GFP^-^/resting CD4^+^ T cells from three donors by qRT-PCR. CEM T cells were used as control. No significant difference in SAMHD1 (**Figure 3D**) nor in APOBEC3G (**Figure 3E**) RNA levels was observed between GFP^-^/resting and GFP^+^ CD4^+^ T cells (*P* = 0.6406 and 0.2969, respectively, *n* = 7). This suggest that the apparent low levels of HIV transcripts observed in GFP^-^/resting CD4^+^ T cells are not due to the action of these restriction factors.

Finally, in order to study the reactivation potential of the lentivirus vectors, infected GFP^-^/resting CD4^+^ T cells were sorted ∼15 days after HSP culture and TCR-stimulated by co-culturing with dendritic cells pulsed with the SEB antigen (SEB-pulsed DCs) or left untreated. Sorted resting CD4^+^ T cells became highly activated and vigorously proliferated during 72 h (**Figure 3F**), however, no increase of GFP^+^ cells could be detected. No significant changes were observed in cells cultured further for 7 days (data not shown). Interestingly, we observed a decrease of GFP transcripts relative to that of EF-1α in TCR-stimulated cells compared to untreated cells (**Figure 3G**). The decrease in relative GFP transcripts after DC/SEB stimulation is likely due to the increase of endogenous cellular mRNA species including the reference gene (EF-1α) after TCR activation. Identical results were obtained by TCR stimulation of HSP-cultured resting CD4^+^ T cells from two additional donors. Taken together, despite the fact that the resting CD4^+^ T cells in HSP culture harbor integrated proviral genomes and express low levels of GFP, LTR-driven mRNA expression is either specifically suppressed by yet unknown mechanisms or remains unresponsive to TCR stimuli.

### Total and Naïve HSP-Cultured CD4^+^ T Cells Support Productive HIV-1_NL-E_ Infection

**FIGURE 4:**
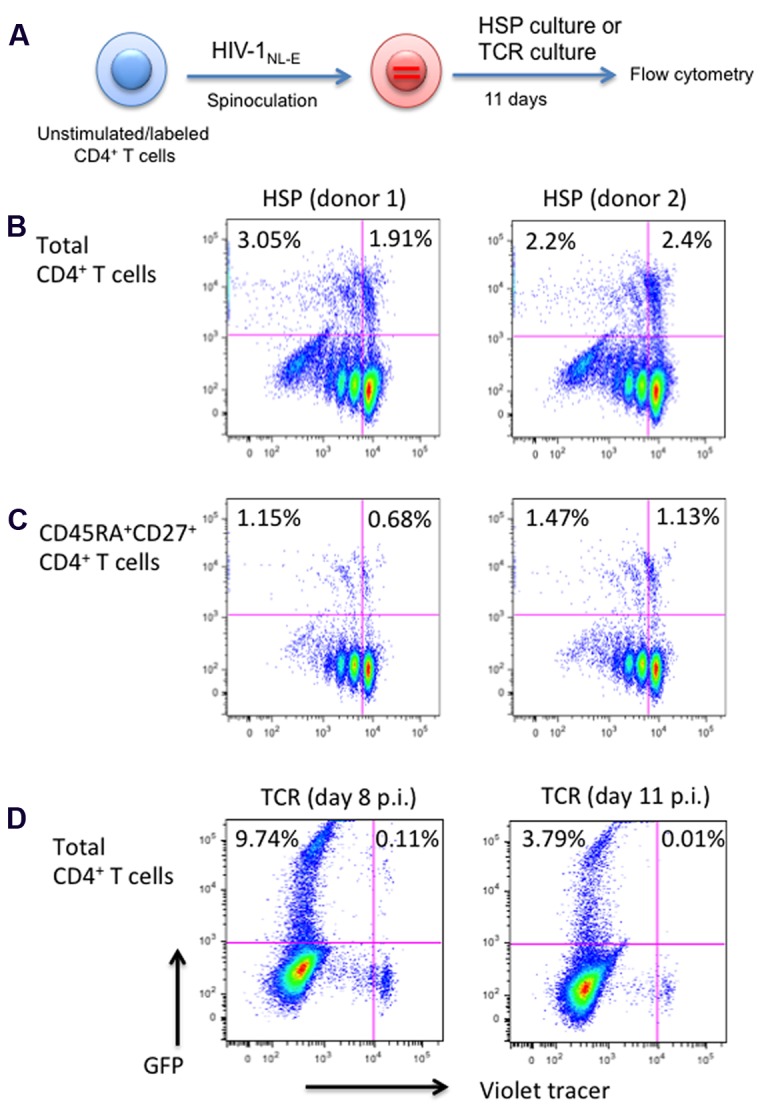
**Total and naïve, HSP-cultured CD4^+^ T cells are both susceptible to productive HIV infection.** **(A)** Outline of experimental strategy. Violet tracer-labeled CD4^+^ T cells from two blood donors were infected with a wild-type HIV-1_NL-E_ and cultured under HSP conditions for 11 days. GFP expression and proliferation were analyzed by flow cytometry. **(B)** Total CD4^+^ T cells. **(C)** CD45RA^+^CD27^+^ naïve CD4^+^ T cells. **(D)** As a control, violet tracer-labeled total CD4^+^ T cells were infected with HIV-1_NL-E_, TCR stimulated with anti-CD3 and anti-CD28, and cultured in the presence of IL-2. Cells were analyzed by flow cytometry at 8 days (left panel) or 11 days (right panel) post infection. Percentages of GFP-positive cells are given for the proliferated and resting cell populations.

The HIV-1 Tat protein is known to regulate the transcriptional activity of the HIV LTR promoter (Donahue et al., 2012). As the Lenti LTR-GFP vector did not encode accessory genes, including *tat*, only the basal promoter activity was assessed in the experiments described in **Figures 2** to **3**. To test the effect of HSP culture conditions in the context of an HIV genome that contains all accessory proteins including Tat, we used the parental HIV-1_NL-E_ construct as shown in **Figure 2A**. Briefly, purified CD4^+^ T cells from two healthy donors were infected with HIV-1_NL-E_ virus at MOI = 0.2, cultured in HSP culture and analyzed for proliferation and GFP expression by flow cytometry at day 11 before virus-induced cytopathic effect (CPE) became apparent (**Figure 4A**). Approximately 5% of total CD4^+^ T cells expressed GFP, with half of these being in a resting state (1.9% in donor 1 and 2.4% in donor 2, **Figure 4B**). Around 50% from the total CD4^+^ T cells corresponded to the naive phenotype (CD45RA^+^CD27^+^, as defined earlier), similar as in **Figure 1B**. When naïve CD4^+^ T cells were gated, 0.68 and 1.13% of resting cells in donor 1 and donor 2, respectively, were GFP^+^ (**Figure 4C**). In HIV-1_NL-E_-infected cells, resting CD4^+^ T cells expressing GFP were more abundant (1.91∼2.4% in ∼5% of total GFP^+^ cells in **Figure 4B**) compared to those in Lenti LTR-GFP-infected cells (0.38% in 3.7% of total GFP^+^ cells in **Figure 3B**). This suggests that the ongoing replication cycles of a wild-type HIV-1_NL-E_ enhances the infectivity of HSP-cultured resting CD4^+^ T cells with both naïve and memory phenotype. It is also possible that the presence of Tat enhanced the GFP expression driven by the LTR promoter in HIV-1-integrated resting CD4^+^ T cells. In contrast, massive HIV-1 infection and higher level of GFP expression were evident in TCR-proliferated CD4^+^ T cells at day 8 p.i. (**Figure 4D**, left) but the frequency of HIV-1-infected cells was substantially decreased at day 11 p.i (**Figure 4D**, right) because of the strong CPE of HIV-1.

### The Latency-Reversing Agent Vorinostat (SAHA) Is Able to Reactivate HIV Provirus in TCR- But Not in HSP-Cultured CD4^+^ T Cells

**FIGURE 5:**
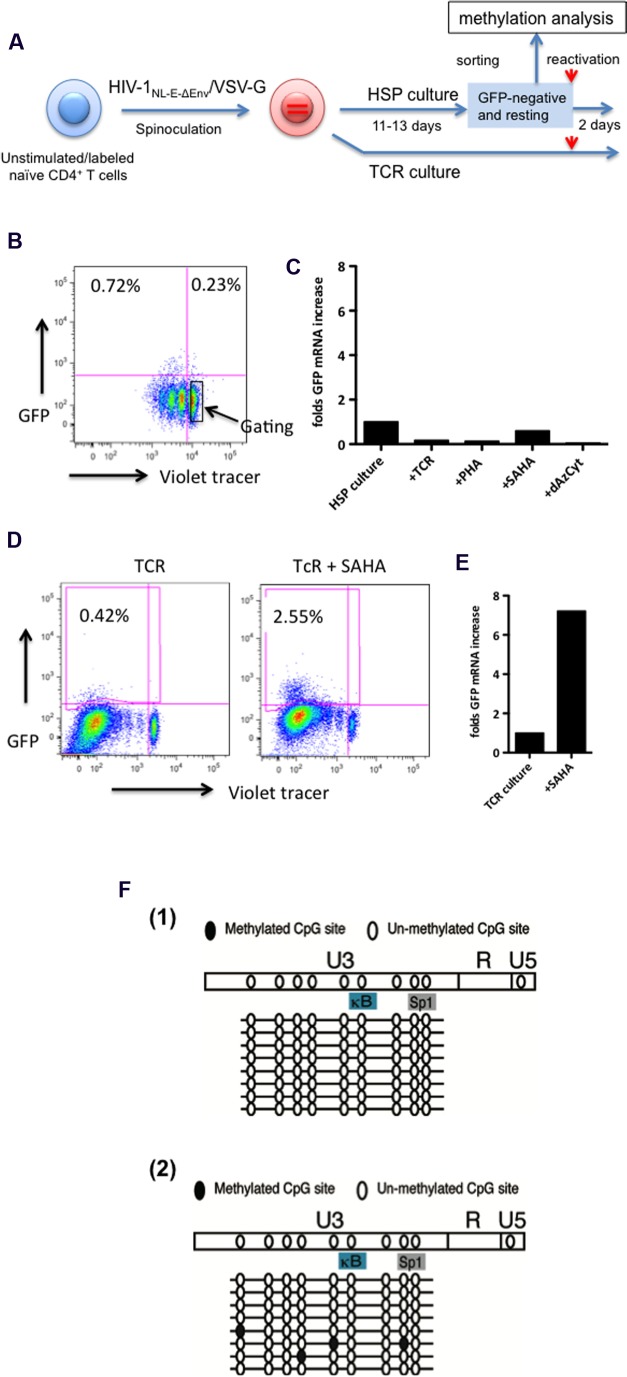
**The HIV-1 provirus is refractory to reactivation in resting, latently infected HSP-cultured CD4^+^ T cells in spite of lack of extensive LTR methylation.** **(A)** Outline of experimental strategy. Violet tracer-labeled, naïve CD4^+^ T cells were infected with VSV-G protein pseudotyped HIV-1_NL-E-ΔEnv_ (HIV-1_NL-E-ΔEnv_/VSV-G) and cultured under HSP or TCR conditions for 11–13 days. GFP-negative, resting cells from the HSP culture (marked in square) were sorted by preparative flow cytometry as shown in the representative dot plots **(B)**. Sorted cells were subjected to various stimuli for 2 days, lysed and the GFP mRNA subjected to quantitative RT-PCR. Transcripts levels were normalized to endogenous RNase P mRNA levels. **(C)** In parallel, cells from the TCR culture were treated with SAHA or left without, and were tested for GFP expression by flow cytometry **(D)**, and for GFP transcript levels by quantitative RT-PCR **(E)**. Sorted cells as in **(B)** from three blood donors were subjected to methylation analysis of the LTR region **(F)**. In two of the three donors, no LTR site was methylated [**F(1)**] while a few sites were methylated in one donor [**F(2)**].

Next, we investigated the effect of various reactivation stimuli on the latent infection, in particular, of resting naïve CD4^+^ T cells maintained under HSP conditions. For this, naïve-enriched CD4^+^ T cells from healthy donors were infected with HIV-1_NL-E-ΔEnv_/VSV-G and cultured for 11–13 days under HSP or TCR conditions (**Figure 5A**). The frequency of total GFP^+^ cells in the pseudotyped HIV_NL-E_ infection was around 1% with only a few positive cells being detectable in the resting naïve CD4^+^T cell fraction (**Figure 5B**, representative of several donors at 11 days post infection). The GFP^-^/resting cells were sorted (**Figure 5B**, enclosed square) and cultured further under HSP conditions or activated either with anti-CD3 and anti-CD28, phytohaemagglutinin (PHA), the HDAC inhibitor Vorinostat (SAHA) or the methylation inhibitor 5-Aza-2′-deoxycytidine (dAzCyt). Both SAHA and dAzCyt are commonly used as reactivators in many *in vitro* HIV latency models (Kauder et al., 2009; Friedman et al., 2011; Ho et al., 2013). Two days after stimulation, total RNA was isolated from the cells and the relative amounts of GFP mRNA were analyzed by qRT-PCR (**Figure 5C**). Here, we chose RNase P as a reference gene because it is known to be a good internal, low copy number mRNA control suitable for qRT-PCR analysis in various tissues (Ikeno et al., 2013). Strikingly, we observed no increase in the relative GFP transcript levels in treated cells compared to untreated cells (**Figure 5C**). This suggests that the reactivation of HIV-1 is not achievable by these strong stimuli in HSP-cultured resting naïve CD4^+^ T cells.

As a control, infected naïve CD4^+^ cells were TCR-stimulated and maintained in the presence of IL-2 for 13 days. These T cells were cultured for additional two days in the absence or presence of SAHA, and GFP expression was analyzed by flow cytometry as well as by qRT-PCR (**Figures 5D,E**, respectively). The representative FACS results of one of two donors are shown in **Figure 5D**. Compared to untreated controls, addition of SAHA resulted in a sixfold increase in the number of GFP^+^ cells (**Figure 5D**), which correlated with a sevenfold increase in the relative GFP mRNA levels (**Figure 5E**). These results are consistent with those reported by others that HIV-1 in resting memory/effector cells that were initially TCR-activated and returned to the resting state could be reactivated by SAHA. Taken together, our results suggest that HIV-1 latency may be maintained in HSP-cultured resting naïve CD4^+^ T cells by a different mechanism from that in TCR-cultured resting memory CD4^+^ T cells.

### Lack of Epigenetic Methylation of the HIV-1 LTR in Resting HSP-Cultured CD4^+^ T Cells

Methylation at the HIV-1 LTR promoter may influence its transcriptional activity. Thus, we analyzed the methylation status of the integrated HIV-1 LTR in sorted resting naïve CD4^+^ T cells of 3 donors cultured under HSP culture conditions (**Figure 5A**). The results are shown in **Figure 5F**. Two donors showed no methylation in the U3 region of the LTR [**Figure 5F(1)**], whereas the remaining donor exhibited methylation only at a few sites [**Figure 5F(2)**]. These results suggest that the methylation status of the LTR is not the cause for the lack of HIV reactivation in resting naïve CD4^+^ T cells.

## Discussion

Perhaps the most striking finding of our work is the observation that HIV-1 proviruses in homeostatically maintained resting naïve CD4^+^ T cells are not reactivated by TCR-mediated signaling. Latently infected naïve T cells represent only a minor component of the total HIV reservoir in infected individuals, however, they have a very long half-life and can feed the pool of infected memory T cells. Identifying the physiological signals under which these cells may produce infectious virus is therefore utterly important for curative therapy strategies. Our data now suggest that there may exist a unique mechanism of blocking provirus reactivation that requires other or additional signals for HIV propagation than in infected memory T cells. There remains the possibility that provirus^+^ T cells are preferentially lost during 24–48 h cultivation especially when massive T cell-proliferation was induced by TCR stimulation as shown in **Figure 3F**. However, in the case of the HDAC inhibitor SAHA, cells were not proliferating, and no increase of GFP protein (data not shown) and GFP mRNA (**Figure 5C**) was observed. In contrast, the same amounts of HIV-loaded CD4^+^ T cells maintained in TCR culture clearly express GFP protein and transcripts after SAHA stimulation (**Figures 5D,E**). As we described in the result of **Figure 3C**, GFP transcripts per one proviral copy in GFP^-^/resting CD4^+^ T cells was calculated to be about fivefold higher than that in GFP^+^ cells. This indicates that a translational block may occur in GFP^-^/resting CD4^+^ T cells. In this line, Mohammadi et al. (2014) suggests that post-transcriptional blocks contribute to latency in their primary cell model. Taken all together, we conclude that provirus containing naïve resting CD4^+^ T cells do not respond to reactivation stimuli with encoded protein expression.

HIV-1 proviruses were refractory to reactivation by the HDAC inhibitor SAHA in the HSP-maintained naïve CD4^+^ T cell fraction. However, recent observations by others suggest that HIV latency in resting CD4^+^ T cells may be reversed through NF-kB signaling (Saleh et al., 2016) or latency reversing agents (Zerbato et al., 2016). These studies are based on resting CD4^+^ T cells pre-treated with the CCR7-ligand CCL19 which activates phosphatidylinositol 3-kinase (PI3K) and phospholipase C (PLC) pathways (Evans et al., 2012) increasing permissiveness of HIV infection in these cells (Saleh et al., 2007). Thus, the reactivation discrepancy between these studies and ours may be partly explained by the fact that their “resting cells” are not necessarily non-dividing cells. In addition, it has been recently shown that HIV integration efficiency is higher in IL-7-treated than in CCL19-treated CD4^+^ T cells (Chavez et al., 2015), and that IL-7-treated CD4^+^ T cells do not induce NFAT or PI3K signaling (Ducrey-Rundquist et al., 2002). Taken together, it seems likely that these different stimuli support distinct intracellular environments that differentially affect HIV-1 infection and latency reversal.

The main drivers of HSP are the cytokines IL-7 and IL-15, which signal through the Janus kinase-signal transducer and activator of transcription pathway (JAK-STAT). This leads to differential expression of several molecules that participate in the regulation of cell proliferation and apoptosis (Mackall et al., 2011; Jabri and Abadie, 2015). Ducrey-Rundquist et al. (2002) speculate about the role of the JAK-STAT pathway for HIV permissiveness in IL-7 treated CD4^+^ T cells. However, whether such cellular factors along the JAK-STAT pathway might be playing a direct role in suppressing LTR-mediated HIV expression cells remains to be explored.

Quantitative reservoir assessment by qPCR demonstrated that the majority of latently infected T cells are within the memory cell population which outnumber infected naïve T cells by about 10-fold (Chomont et al., 2009; Josefsson et al., 2013a,b). Nonetheless, naïve T cells have a very long half-life and can survive as resting cells for extended periods of time (McCune et al., 2000; Vrisekoop et al., 2008). Furthermore, they are also maintained by low-level activation and HSP (Surh and Sprent, 2008). This low activation level may translate to a low level of HIV expression that is insufficient for dying through virus-mediated CPEs and immune surveillance but sufficient for feeding the pool of latently and productively infected cells. Indeed, the HIV burst size of experimentally infected naïve T cells reincorporated into a human tonsil culture was about eightfold lower than that of memory T cells (Eckstein et al., 2001). In addition, HIV-producing naïve CD4^+^ T cells have been detected in lymph nodes by *in situ* hybridization (Zhang et al., 1999), and, under special conditions of an acute SHIV infection of macaques, naïve CD4^+^T cells represent the majority of infected CD4^+^T cells (Nishimura et al., 2009). Thus, HIV-1-infected naïve T cells represent a relevant component of the latent reservoir.

Current *in vitro* HIV latency-reactivation models are mainly based on TCR-stimulated resting memory/effector CD4^+^ T cells (Sahu et al., 2006; Marini et al., 2008; Tyagi et al., 2010), and the reactivation of latent HIV-1 in resting naïve CD4^+^ T cells undergoing HSP has not been further investigated. Here, we describe an *in vitro* culture system of HSP that allows studying HIV-1 latency in naïve CD4^+^ T cells. We show that cells infected with a GFP-encoding HIV-1 and cultured under HSP conditions were maintained in a non-dividing ( = resting) state. Only a small fraction of GFP^+^ cells were detected compared to those observed in TCR-stimulated memory cells despite that HIV-1 integration and GFP mRNA was detectable in naïve CD4^+^ T cells. Interestingly, neither TCR nor chemical stimuli like SAHA were able to reactivate HIV-1 provirus in HSP-cultured naïve CD4^+^ T cells. Our results suggest that this lack of activity is not due to an increased expression of the restriction factors SAMHD1 or APOBEC3G, nor to the methylation status of the LTR. These observations are in agreement with a recent report by Coiras et al. (2016) showing that IL-7 treatment of CD4^+^ T cells induces SAMHD1 phosphorylation abrogating its antiviral activity. Although additional epigenetic regulation cannot be ruled out, altogether these results point to a mechanism of HIV control in resting naïve CD4^+^ T cells that may be distinct from that in TCR-stimulated memory/effector CD4^+^ T cells.

Antigen-dependent TCR activation of CD4^+^ T cells is the most efficient stimulus for productive HIV-1 infection *in vivo*. In an antigen-independent culture system containing IL-2, Woods et al. reported that CD45RA^+^ naïve CD4^+^ T cells easily lose HIV-1 productivity after entry (Woods et al., 1997) suggesting that HIV-1 requires cellular activation signals beyond that provided by IL-2 alone. It has been argued that HIV-1 enter resting naïve cells less efficiently than memory cells (Dai et al., 2009), and that VSV-pseudotyped HIV cannot fuse or establish latent infection in unstimulated, resting CD4 T cells (Agosto et al., 2009; Yu et al., 2009). However, other studies have shown that VSV-pseudotyped HIV was able to integrate into both naïve and memory resting CD4^+^ T cells mixed with dendritic cells at similar efficiencies (Dai et al., 2009). While we did not discriminate the Lenti LTR-GFP integration kinetics between naïve and memory T cell infection, we show here that naïve cells were able to express a substantial level of viral RNA even 2 weeks after VSV-pseudotyped HIV-1 infection. This indicates that the infection process of resting naïve CD4^+^ T cells successfully progressed by cytokine stimulation immediately after spinoculation. However, whether antigen-independent activation by cytokines favorably supports latent HIV infection or permanently suppresses integrated HIV-1 remains to be addressed.

In summary, here we show that HIV-1 integration and transcription were detectable in resting naïve CD4^+^ T cells cultured under HSP. HSP-cultured cells slowly proliferated and maintained their naïve phenotype. Interestingly, latently infected resting naïve cells kept under these conditions were reactivation resistant. The nature of this apparent lack of reactivation is unknown but may relate to stochasticity of HIV promoter activation or present a distinct mechanism of provirus silencing related to the HSP maintenance process in different T cell subsets. The latter seems to be reinforced by a recent report suggesting that the efficiency of latency reversing agents may be T cell phenotype-dependent (Baxter et al., 2016). Currently, however, we cannot eliminate the possibility that all proviral DNAs in naïve resting CD4^+^ T cells exist as defective proviruses and, therefore, will never be replication-competent. Altogether, given the contribution of naïve CD4^+^ T cells to the latent HIV reservoir *in vivo*, our results and those of others, warrant further studies on HIV reactivation in latently infected cells that are maintained by homeostatic mechanisms.

## Ethics Statement

We called for healthy volunteers for donating blood and explained our experiments. Blood was taken from these donors after obtaining the written informed consent according to the format provided by the Ethical Committee for the medical research using human resources in the National Institute of Infectious Diseases (NIID, Tokyo, Japan).

## Author Contributions

YT-Y designed the project and together with JM and AM wrote the manuscript. YT-Y, KTo, HT, JM, and AM discussed the results. YT-Y performed experiments with the help of MK-I, YW, KTe, AK-T, MY and JM. All authors reviewed the manuscript.

## Conflict of Interest Statement

The authors declare that the research was conducted in the absence of any commercial or financial relationships that could be construed as a potential conflict of interest.
